# The association between dental and dentoalveolar arch forms of children with normal occlusion and malocclusion: a cross-sectional study

**DOI:** 10.1186/s12903-024-04515-z

**Published:** 2024-06-25

**Authors:** Jiaqing Luo, Taiqi Liu, Yi Wang, Xiaobing Li

**Affiliations:** 1 School of Computer Science and Engineering, University of Electronic Science and Technology of China, No. 2006, Xiyuan Ave, West Hi-Tech Zone, Chengdu, Sichuan China 611731; 2 Supalign (Chengdu) Technology Co. Ltd, No. 531, Building 2, No. 33, Wuqing South Road, Chengdu, Sichuan 610046 China; 3 State Key Laboratory of Oral Diseases, National Clinical Research Center for Oral Diseases, West China Hospital of Stomatology, Sichuan University, No. 14, People’s South Road, Chengdu, Sichuan 610041 China; 4 Department of Pediatric Dentistry, West China Hospital of Stomatology, Sichuan University, No. 14, People’s South Road, Chengdu, Sichuan 610041 China

**Keywords:** Orthodontics, Interceptive, Cluster analyses, Dental arches, Alveolar process

## Abstract

**Background:**

Symmetrical and coordinated dental and alveolar arches are crucial for achieving proper occlusion. This study aimed to explore the association between dental and dentoalveolar arch forms in children with both normal occlusion and malocclusion.

**Methods:**

209 normal occlusion subjects (5–13 years, mean 8.48 years) and 199 malocclusion subjects (5–12 years, mean 8.19 years) were included. The dentoalveolar arch form was characterized by the smoothest projected curve representing the layered contour of the buccal alveolar bone, referred to as the *LiLo* curve. Subsequently, a polynomial function was utilized to assess dental and dentoalveolar arch forms. To facilitate separate analyses of shape (depth/width ratio) and size (depth and width), the widths of dental and dentoalveolar arch forms were normalized. The normalized dental and dentoalveolar arch forms (shapes) were further classified into 6 groups, termed *dental/dentoalveolar arch clusters,* using the k-means algorithm.

**Results:**

The association between dental and dentoalveolar arch clusters was found to be one-to-many rather than one-to-one. The mismatch between dental and dentoalveolar arch forms is common in malocclusion, affecting 11.4% of the maxilla and 9.2% of the mandible, respectively.

**Conclusions:**

There are large individual variations in the association between dental and dentoalveolar arch forms. Early orthodontic treatment may play an active role in coordinating the relationship between the dental and dentoalveolar arch forms.

## Introduction

Understanding the association between dental and dentoalveolar arch forms holds paramount importance in devising effective orthodontic treatment plans and ensuring long-term occlusal stability [1]. Many studies attempted to find *individualized* arch forms through WALA ridge/basal arch form, which is an anatomic guide for positioning teeth [1–5], because of the reduced change and limited expansion of the dental arch form in adults [1, 6]. Clinically, it is important to keep the dental arch form unchanged during orthodontic treatment, because occlusal stability depends on the preservation of the patient's original dental arch form [7, 8]. Sanin determined the length and shape of the dental arch form through mathematical functions such as exponential, logarithmic, elliptical, parabolic, hyperbolic, or polynomial [9]. Chuck proposed the first classification of dental arch forms, includes 3 forms: ovoid, tapered, and square shape [10]. The most popular were the Ricketts pentamorphic arch forms, which classified dental arch forms into in 5 forms (i.e., normal, ovoid, tapered, narrow ovoid, and narrow tapered) based on factors such as arch correlation, depth, and length [11, 12].

On the other hand, the malocclusion in permanent dentition is largely due to the abnormal occlusion development in deciduous or mixed dentition [13]. Early orthodontic treatment has the potential to influence the growth of dental and dentoalveolar arch forms, thus preventing the occurrence of malocclusion or reducing the severity of malocclusion. Many studies have investigated the changes in dental arch forms during each stage of growth and development [6, 14–18]. Several cephalometric studies have shown racial variations in facial traits and changes in dentofacial patterns during periods of active growth [19–21]. A longitudinal study of a Swedish population (5–31 years of age) with normal occlusion showed that the facial pattern changed at different developmental stages, with accelerated growth not only between 13- and 16-year but also between 5- and 7-year [22].

The purpose of this study was to investigate the typical shapes of dental and dentoalveolar arch forms, along with their common associations, to aid in the early detection of morphological anomalies in malocclusion. We hypothesized that there is a quantifiable association between the shapes of dental and dentoalveolar arch forms in children with normal occlusion. This association could serve as a reference for determining the extent of dental/dentoalveolar arch remodeling, including widening, lengthening, and reshaping, in early orthodontic treatment. To address ethical concerns of acquiring Cone-Beam Computed Tomography (CBCT) images from children with normal occlusion, we chose to analyze 3D point clouds instead. Additionally, we proposed a systematic approach to the computer-aided analysis of dental and dentoalveolar arch forms.

## Materials and Methods

### Participants

The normal occlusion group is a sample of 209 (103 males, 106 females, 5 to 13 years, mean age 8.48 years, standard deviation 1.98 years) selected from 2,056 school-age children in Chengdu, China, between Jan 2021 and Jan 2022. These subjects responded to a community oral health sampling survey conducted by the West China Hospital of Stomatology, Sichuan University. Previous study has shown that the proportion of normal occlusion is only about 11% [23]. The sample size calculation was based on cross-sectional survey studies using sample size charts with a power of 0.95. The minimum number of subjects expected to be included in this study was 146. 209 of 2056 subjects fulfilled the following inclusion criteria: 1) mixed and early permanent dentition; 2) aligned dentition with normal occlusive relationship; 3) canine and molar Class I relationship: Class I soft-tissue, proximately flat maxillary and mandible occlusal curves, minimal crowding (< 2 mm) and space (< 1 mm), normal overjet (approximately 2–4 mm) and overbite (upper incisors cover less than 1/3 of the lower incisors); 4) symmetrical and normal facial growth patterns; 5) normal tooth numbers and tooth replacements.

The malocclusion group contains 199 samples (64 males, 135 females, 5 to 12 years, mean age 8.19 years, standard deviation 1.29 years) selected from the department of Pediatric dentistry of the West China Hospital of Stomatology, Sichuan University. The inclusion criteria were: 1) mixed and early permanent dentition; 2) malocclusive relationships of teeth (determined through lateral cephalograms): 91 cases were identified with skeletal Class I (0° < ANB < 5°) malocclusion, 81 cases exhibited skeletal Class II (ANB > 5°) malocclusion, and 27 cases presented skeletal Class III (ANB < 0°) malocclusion; 3) transverse or sagittal dental arch deficiency (narrow dental arch or shortened dental arch length).

The exclusion criteria for both groups included: 1) systemic or genetic diseases, upper airway diseases; 2) obvious teeth abrasion, attrition and deformity; 3) history of orthodontic, prosthodontic treatments and dentofacial surgery; 4) incomplete dental cast.

### Framework

**Fig. 1:**
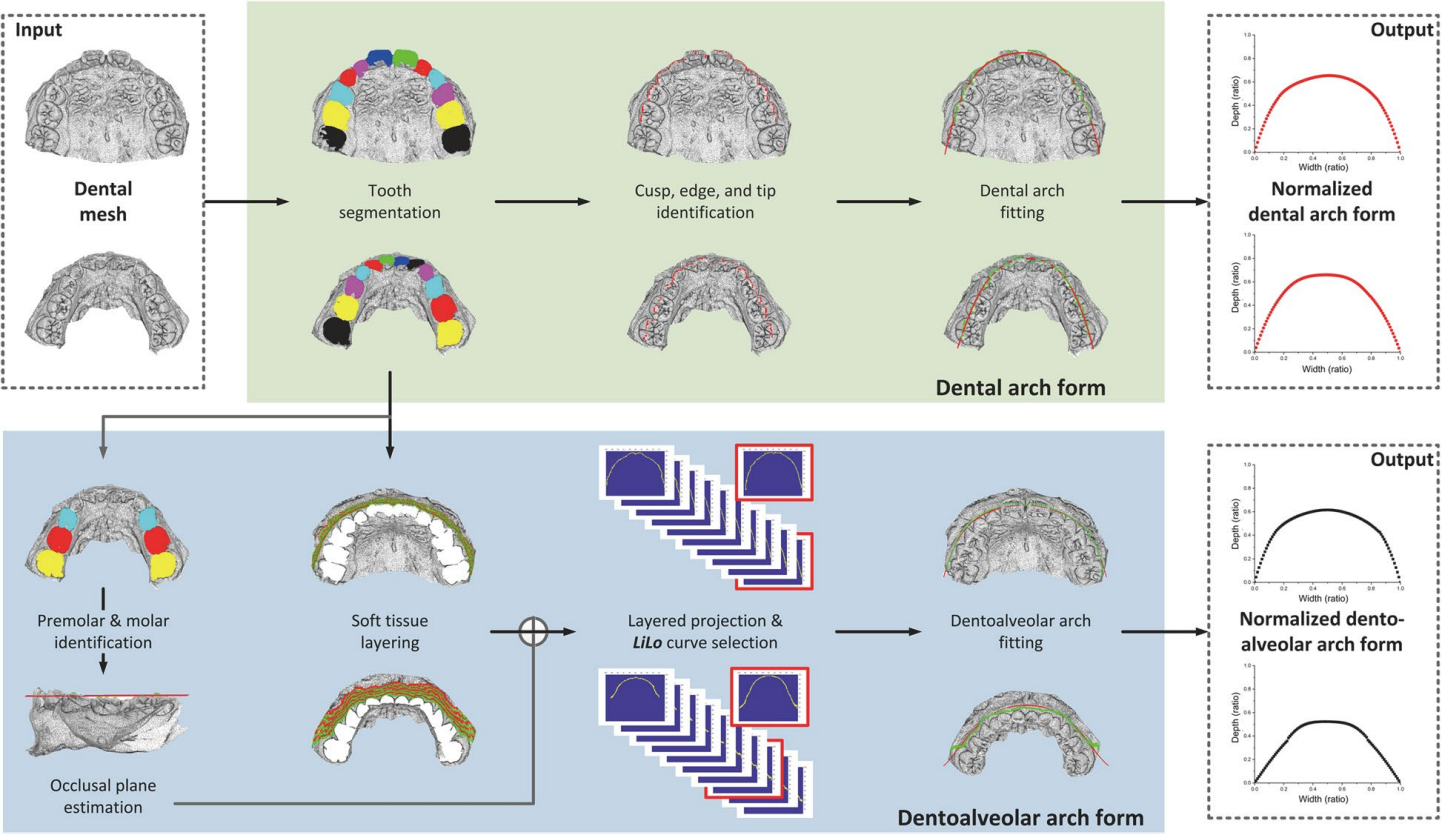
The system framework and the data processing flow. The input is the dental mesh and the outputs are normalized dental and dentoalveolar arch forms

We present a novel framework (Fig. 1) for the automatic identification of dental and dentoalveolar arch forms. Our approach takes the 3D scanned point cloud data of dental casts as input and normalized dental and dentoalveolar arch forms as output.

Dental casts were scanned using a UP3D laser scanner (model UP400) with an accuracy of 8 μm. The following data analysis, visualization, and numerical calculations used MATLAB (version 2017a (9.2.0538062)).

The identification of dental arch form includes 3 steps. 1) *Tooth segmentation:* we design an automatic method to extract the individual tooth from dental mesh by curvature and contour line [24–27]. 2) *Cusp, tip, and edge identification:* we identify the buccal cusps of molars and premolar, cusp tips of canines, and incisal edges of incisors by features such as curvature and height. 3) *Dental arch fitting:* we use a 6th degree polynomial function [28] to generate a fitting curve for the buccal cusps of molars and premolars, cusp tips of canines, and incisal edges of incisors.

The identification of the dentoalveolar arch form consists of 4 steps. 1) *Occlusal plane estimation:* we first identify the mandibular 1st and 2nd primary molars (or 1st and 2nd premolars), 1st permanent molar, and then find the best-fit plane through the cusp tips of those teeth. 2) *Soft tissue layering:* we remove the teeth from the 3D dental mesh and divide the soft tissue from the gingival margin to the alveolar mucosa into 10 layers. 3) *LiLo curve selection:* we project the soft tissue layers onto the occlusal plane, and then select the smoothest curve by the standard deviation of the first difference. 4) *Dentoalveolar arch fitting:* we use a 6th degree polynomial function to generate the fitting curve for the soft tissue layers corresponding to the *LiLo* curve.

### Measurements

By considering both clinical and engineering practice, we defined the terms used in the framework as follows:

1. Dental arch form: the fitting curve of the buccal cusps.
2. Occlusal plane: the best-fit plane through the cusp tips of mandibular 1st and 2nd premolars, and 1st permanent molar. The 3D dental mesh is rotated so the transverse plane (*XOY* plane) coincided with the occlusal plane.
3. *LiLo* curve: the soft tissue from the gingival margin to the alveolar mucosa is divided into *n* layers (e.g., *n* = 10 in Fig. 1). Each layer is projected onto the occlusal plane, and the smoothest projection curve is called the *LiLo* curve. The reason for choosing the smoothest curve was to avoid capturing the gingival margin, which may result in many corner points after projection. Although the *LiLo* curve is not an anatomical curve, it reflects the characteristics of the dentoalveolar arch form. A meaningful and acceptable metric may facilitate academic collaboration across different communities and scientific fields [29].
4. Dentoalveolar arch form: the fitting curve of the soft tissue layer corresponding to the *LiLo* curve.
5. Mid-sagittal line: the sagittal line through interproximal contact point of the central incisors. The midsagittal plane (*YOZ* plane) is defined as passing the mid-sagittal line and perpendicular to the occlusal plane.
6. Distal-transverse line: the transverse line tangent to the distal end of the molar crown of the dental arch.
7. Arch depth (measurement of *y*): the distance from the intersection point of the mid-sagittal line and the dental/dentoalveolar arch form to the intersection point of the mid-sagittal line and the distal-transverse line.
8. Arch width (measurement of *x*): the distance between two intersection points of the distal-transverse line and the dental/dentoalveolar arch form.
9. Normalized arch form: the 2D shape (depth/width ratio) of the dental/dentoalveolar arch form defined in *X*-axis and *Y*-axis The arch width is scaled to 1, i.e., *x* is normalized to the range in [0, 1] by max–min normalization. The normalized *x*, *x′*, can be given by:
  1 $$x{\prime}=\frac{x-min(x)}{max(x)-min(x)}$$

The arch depth is scaled according to the ratio of arch depth to arch width, i.e., *y* is scaled to the range in $[0,\frac{max(y)-min(y)}{max(x)-min(x)}]$. The scaled *y*, *y′*, can be written as:

2 $$y{\prime}=\frac{y-min(y)}{max(x)-min(x)}$$

### Algorithms

A *classifier* is a type of machine learning algorithm used to train a classification model to predict the class of a target. The normalized dental and dentoalveolar arch forms with normal occlusions were compared by 3 classifiers: K-Nearest Neighbors (KNN), Support Vector Machines (SVM) and Naive Bayes (NB) [30].

The relevant quantities for calculating the metrics for a binary classifier are the 4 entries in the confusion matrix.

3 $$M=\left[\begin{array}{cc}TP& FN\\ FP& TN\end{array}\right]$$

True positive (TP): the number of correctly classified positive samples.

True negative (TN): the number of correctly classified negative samples.

False positive (FP): the number of samples incorrectly classified as positive.

False negative (FN): the number of samples incorrectly classified as negative.

The *accuracy* is the ratio between the correctly classified samples and the total number of samples.

4 $$accuracy=\frac{TP+TN}{TP+TN+FP+FN}$$

The *sensitivity* is the rate of positive samples correctly classified.

5 $$sensitivity=\frac{TP}{TP+FN}$$

The *F1 score* is the harmonic mean of precision and sensitivity.

6 $$F1=\frac{2\times TP}{2\times TP+FP+FN}$$

Accuracy is a determining factor when evaluating the accuracy of a machine learning project, especially in healthcare. The higher the accuracy, the better the model performs. For binary classification, an accuracy close to 0.5 indicates difficulty in discrimination (no better than random guessing), and an accuracy close to 1 indicates a discernible difference (for a balanced dataset).

The *k-means* algorithm is one of the most common unsupervised machine learning algorithms for partitioning a given data set into k clusters in which each data point belongs to the cluster with the nearest mean. The normalized dental/dentoalveolar arch forms with normal occlusion were classified into a number of different groups by k-means.

The optimal k in k-means was determined by the Elbow method [31]. The Within-Cluster-Sum of Squared Errors (WSS) was calculated for different values of k, and then the k for which WSS became first starts to diminish was selected by the Cumulative Sum (CuSum) algorithm [32].

The k-means may produce unstable clustering due to factors, such as the initialization of the center and the number of iterations, etc. This instability may lead to bias in detecting morphological anomalies, thus requiring multiple runs with different initializations.

## Results

### Difference

There were 2 types of data, normalized dental arch form (ND) and normalized dentoalveolar arch form (NA). The comparative analysis of ND and NA was divided into 3 steps. 1) *Dataset generation:* the control group had only 1 type of data, while the experimental group contained 2 types of data. 3 datasets were generated for 2 control groups and 1 experimental group. The indices of 209 subjects with normal occlusion were randomly divided into 2 subsets (50% for each subset): S1 and S2. In 2 datasets of control groups (ND–ND and NA-NA), both S1 and S2 were either all ND or all NA. In the dataset of the experimental group (ND-NA), S1 was ND and S2 was NA. 2) *Model training:* 3 classifiers (KNN, SVM and NB) were used for binary classification of 3 datasets (ND–ND, NA-NA, and ND-NA). The indices of subjects were shuffled and then used for the train-test split. The first 80% went to the training set and the last 20% to the testing set. As a result, the accuracy of 9 models was calculated and recorded. 3) *Repeated observations:* the goal of the analysis was not to find the best predictive model but to evaluate the differences between the control and experimental groups. Steps 1) and 2) were repeated 100 times. The average accuracy of different models from each classifier was calculated.

Table 1 depicts that the average accuracy on ND-NA was much higher than that on ND–ND or NA-NA, regardless of which classifier was used. Taking KNN as an example, KNN achieved an average accuracy of 0.838 on the mandibular ND-NA, but only 0.493 and 0.564 on the mandibular ND–ND and NA-NA, respectively. This result indicates that the difference between ND and NA was greater than the difference within ND or NA. Another interesting observation is that the average accuracy on ND-NA is significantly higher than 0.5, which indicates that the classifier can distinguish ND and NA to a certain extent. For example, SVM had an average accuracy of 0.841 and 0.886 for maxilla and mandible on ND-NA, respectively.

**Table 1:** The comparative analysis between normalized dental arch form (ND) and normalized dentoalveolar arch form (NA). 2 datasets (ND–ND and NA-NA) were generated for control groups and 1 dataset (ND-NA) was generated for the experimental group

Parts	Datasets	Classifiers	Average accuracy	95% confidence interval		Average sensitivity	Average F1 score
				Lower	Upper		
Maxilla	ND–ND	KNN	0.53361	0.53238	0.53483	0.54067	0.4999
		NB	0.53309	0.53194	0.53424	0.52695	0.58808
		SVM	0.58641	0.58511	0.58772	0.61855	0.55845
	NA-NA	KNN	0.56733	0.56621	0.56844	0.58301	0.52526
		NB	0.52716	0.52598	0.52833	0.52182	0.59015
		SVM	0.52015	0.51891	0.52139	0.52475	0.56188
	**ND-NA**	KNN	0.7796	0.77879	0.7804	0.76662	0.78376
		NB	0.70821	0.7072	0.70921	0.68742	0.72082
		SVM	**0.84106**	0.84028	0.84184	0.81483	0.84616
Mandible	ND–ND	KNN	0.49373	0.49255	0.49492	0.49365	0.43072
		NB	0.53775	0.53632	0.53918	0.52756	0.60778
		SVM	0.4842	0.48264	0.48576	0.49093	0.49657
	NA-NA	KNN	0.5636	0.56233	0.56486	0.57357	0.5341
		NB	0.61966	0.61849	0.62083	0.59179	0.66937
		SVM	0.58	0.57881	0.58119	0.57164	0.6178
	**ND-NA**	KNN	0.83788	0.83714	0.83862	0.82652	0.83935
		NB	0.86475	0.86403	0.86546	0.83993	0.86835
		SVM	**0.88645**	0.8858	0.88709	0.87975	0.88643

### Association

**Fig. 2:**
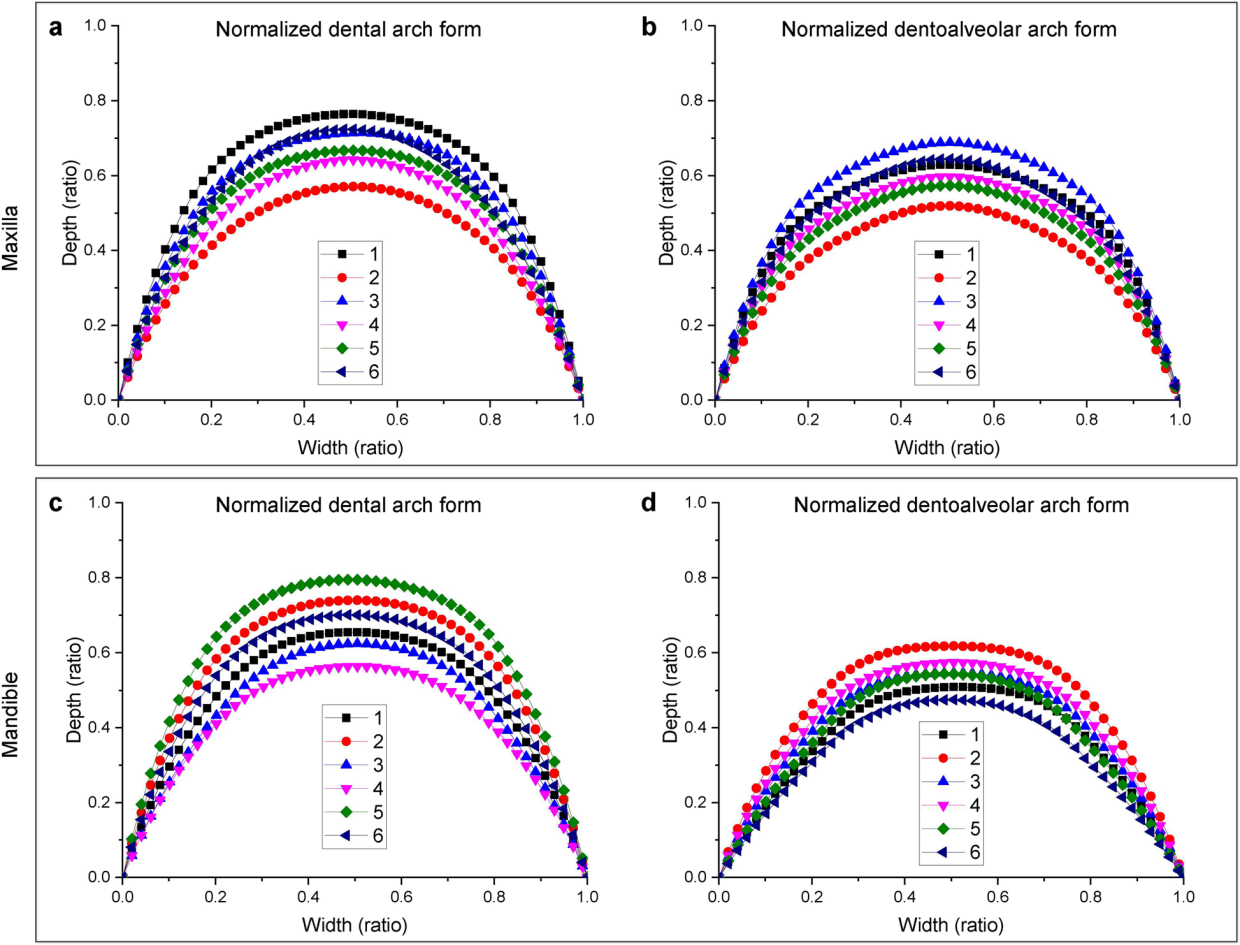
The centers (typical in the normal cluster) of the dental and dentoalveolar arch clusters. The maxillary dental (**a**) and dentoalveolar (**b**) arch cluster centers. The mandibular dental (**c**) and dentoalveolar (**d**) arch cluster centers. Numbers 1–6 in a-d represent the cluster numbers

**Fig. 3:**
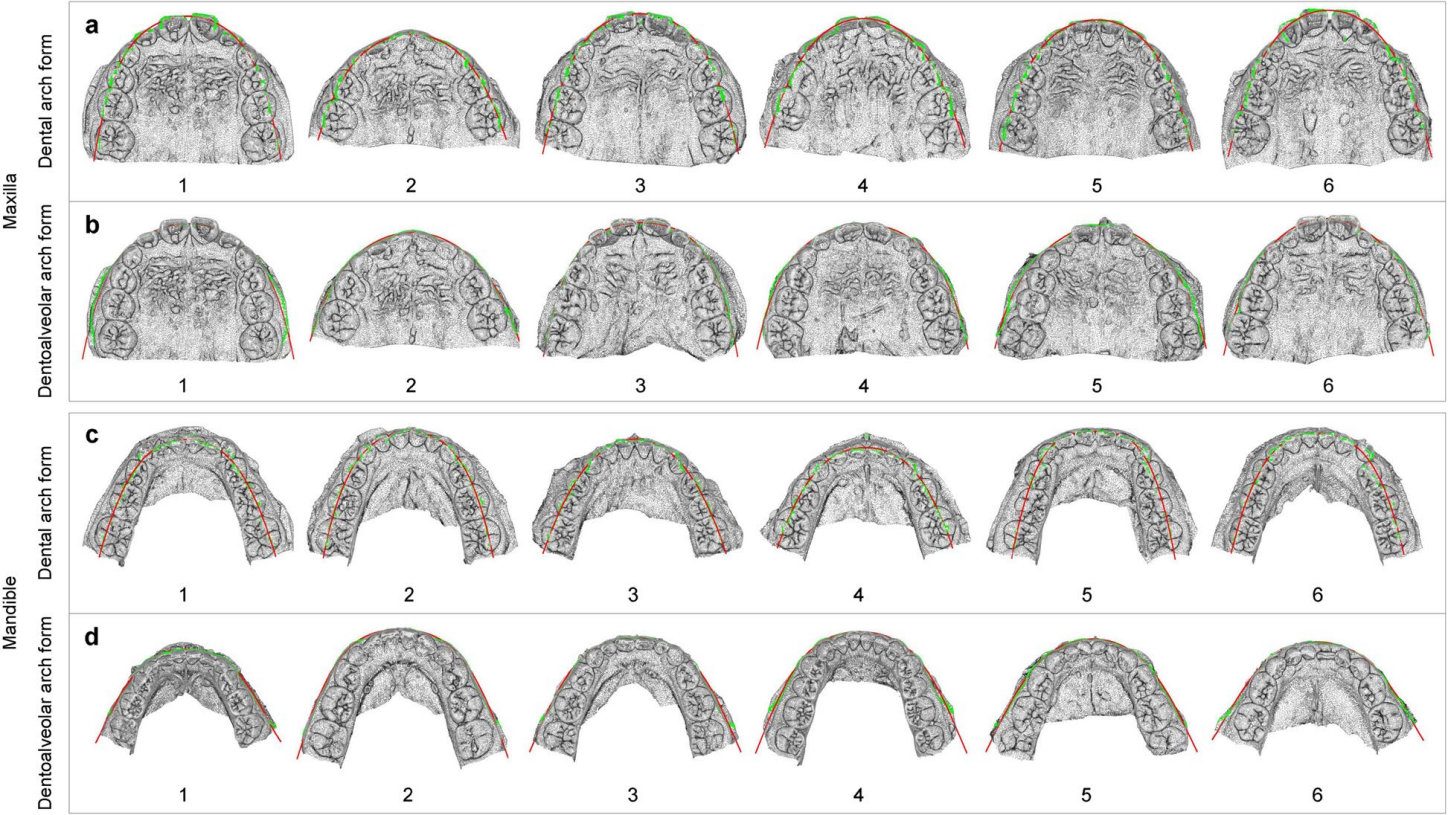
The 3D models (typical samples in the normal cluster) with the normalized dental and dentoalveolar arch forms closest to the cluster centers shown in Fig. 2a-d. The maxillary dental (**a**) and dentoalveolar (b) arch forms. The mandibular dental (**c**) and dentoalveolar (**d**) arch forms. The green dots in (**a**) and (**c**) represent the dental arch landmarks, while that in (**b**) and (**d**) represent the dentoalveolar arch landmarks. The red dotted curves in (**a**) and (**c**) denote the dental arch forms, and that in (**b**) and (**d**) denote the dentoalveolar arch forms

The normalized dental/dentoalveolar arch forms (shapes) of 209 subjects with normal occlusion were divided into different groups, called dental/dentoalveolar arch *clusters*, by the k-means algorithm. 6 dental and 6 dentoalveolar arch clusters were present in both the maxilla (Fig. 2a-b) and mandible (Fig. 2c-d). Each cluster was represented by its center, which can be thought of as the multi-dimensional average of the cluster. Figure 3a-b shows the maxillary 3D models with the normalized dental/dentoalveolar arch forms closest to the dental/dentoalveolar arch cluster centers shown in Fig. 2a-b. These maxillary 3D models reflect the characteristics of the typical subjects for each cluster. The cluster analysis for the mandible was similar to that for the maxilla, and the results are shown in Fig. 2c-d and Fig. 3c-d.

**Fig. 4:**
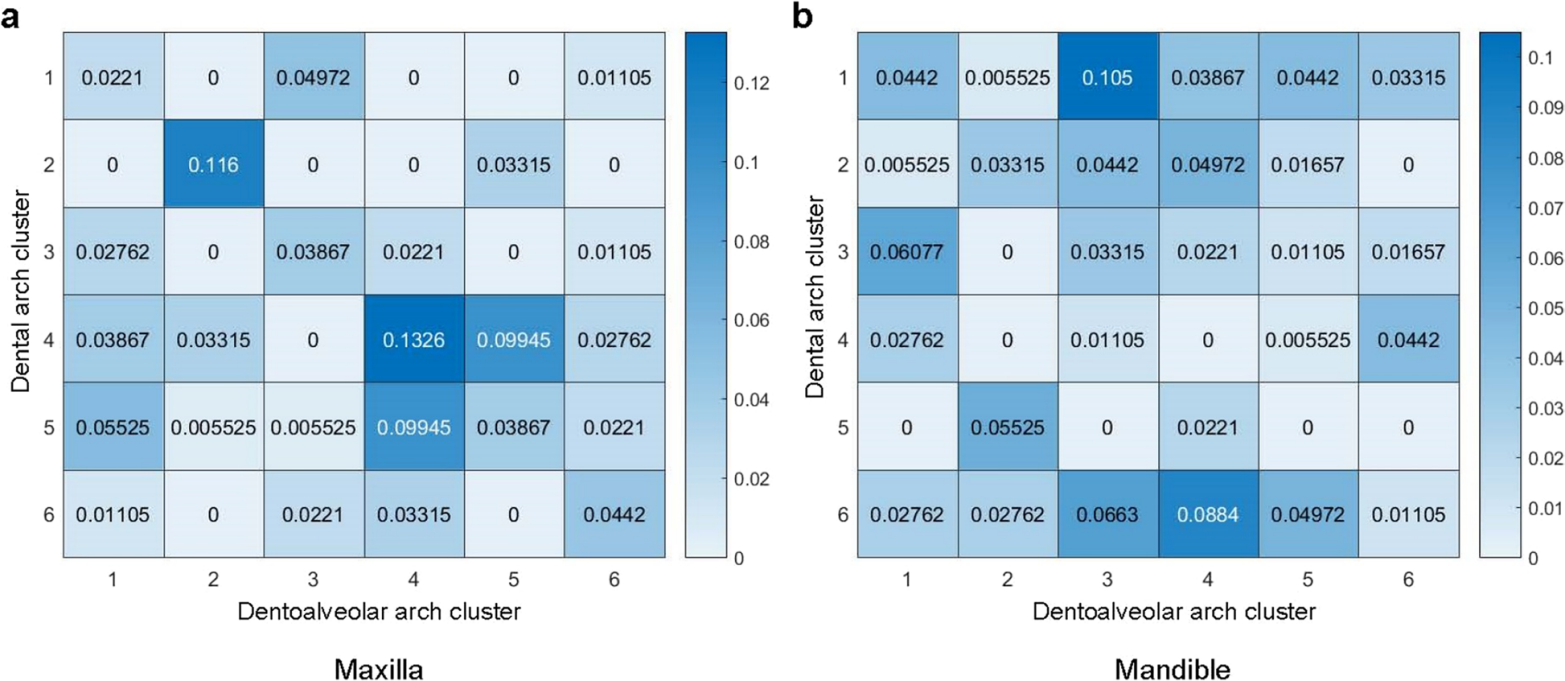
The association between dental and dentoalveolar arch clusters in the maxilla (**a**) and mandible (**b**). Each cell (**i**, **j**) in the heatmap represents the proportion of subjects with normal occlusion that belong to dental arch cluster i and dentoalveolar arch cluster j

The association between dental and dentoalveolar arch clusters is represented by a heatmap in Fig. 4. The rows and columns of the heatmap are dental and dentoalveolar arch clusters, respectively. Each cell(*i*, *j*) in the heatmap was defined as the number of subjects belonging to dental arch cluster *i* and dentoalveolar arch cluster *j* divided by the total number of subjects. Figure 4 indicates that the association between dental and dentoalveolar arch clusters was one-to-many rather than one-to-one. The most common associations of maxillary dental and dentoalveolar arch clusters are (4, 4), (2, 2), and (4, 5), accounting for 0.133, 0.116, and 0.099, respectively (Fig. 4a). The most common associations of mandibular dental and dentoalveolar  arch clusters are (1, 3), (6, 4), and (6, 3), accounting for 0.105, 0.088, and 0.066, respectively (Fig. 4b).

It should be noted that the above analysis illustrates the results of a single run of the modified k-means algorithm, where the optimal k is automatically determined. However, these results may not necessarily depict the "optimal” clustering, which depends on various factors, including algorithm selection, data quality, initialization method, and parameter settings.

### Anomalies

Different types of morphological anomalies were defined and counted. The “*morphological anomaly*” here was a *coarse-grained* definition that focused only on shapes (normalized dental/dentoalveolar forms) and not on dimensions and details. Given normalized dental and dentoalveolar arch forms, *f*_*d*_ and *f*_*a*_, 3 basic anomalies were defined as follows:

1. Abnormal dental arch form (AD): Suppose *C*_*d*_ is the dental arch cluster with the center *c*_*d*_ closest to *f*_*d*_. Let *d*_*d*_ be the Euclidean distance from a member of *C*_*d*_ to *c*_*d*_. The mean *μ*_*d*_ and standard deviation *σ*_*d*_ of all *d*_*d*_ are calculated. If the Euclidean distance from *f*_*d*_ to *c*_*d*_ falls outside the range of *μ*_*d*_ ± 1.96*σ*_*d*_, *f*_*d*_ is considered an outlier.
2. Abnormal dentoalveolar arch form (AA): Suppose *C*_*a*_ is the dentoalveolar arch cluster with the center *c*_*a*_ closest to *f*_*a*_. Let *d*_*a*_ be the Euclidean distance from a member of *C*_*a*_ to *c*_*a*_. The mean *μ*_*a*_ and standard deviation *σ*_*a*_ of all *d*_*a*_ are calculated. If the Euclidean distance from *f*_*a*_ to *c*_*a*_ falls outside the range of *μ*_*a*_ ± 1.96*σ*_*a*_, *f*_*a*_ is considered an outlier.
3. Mismatched dental and dentoalveolar arch forms (MDA): If the value of cell (*C*_*d*_, *C*_*a*_) is close to 0 in Fig. 4 (i.e., (*C*_*d*_, *C*_*a*_) is an unusual morphological association), *f*_*d*_ and *f*_*a*_ are mismatched.

**Fig. 5:**
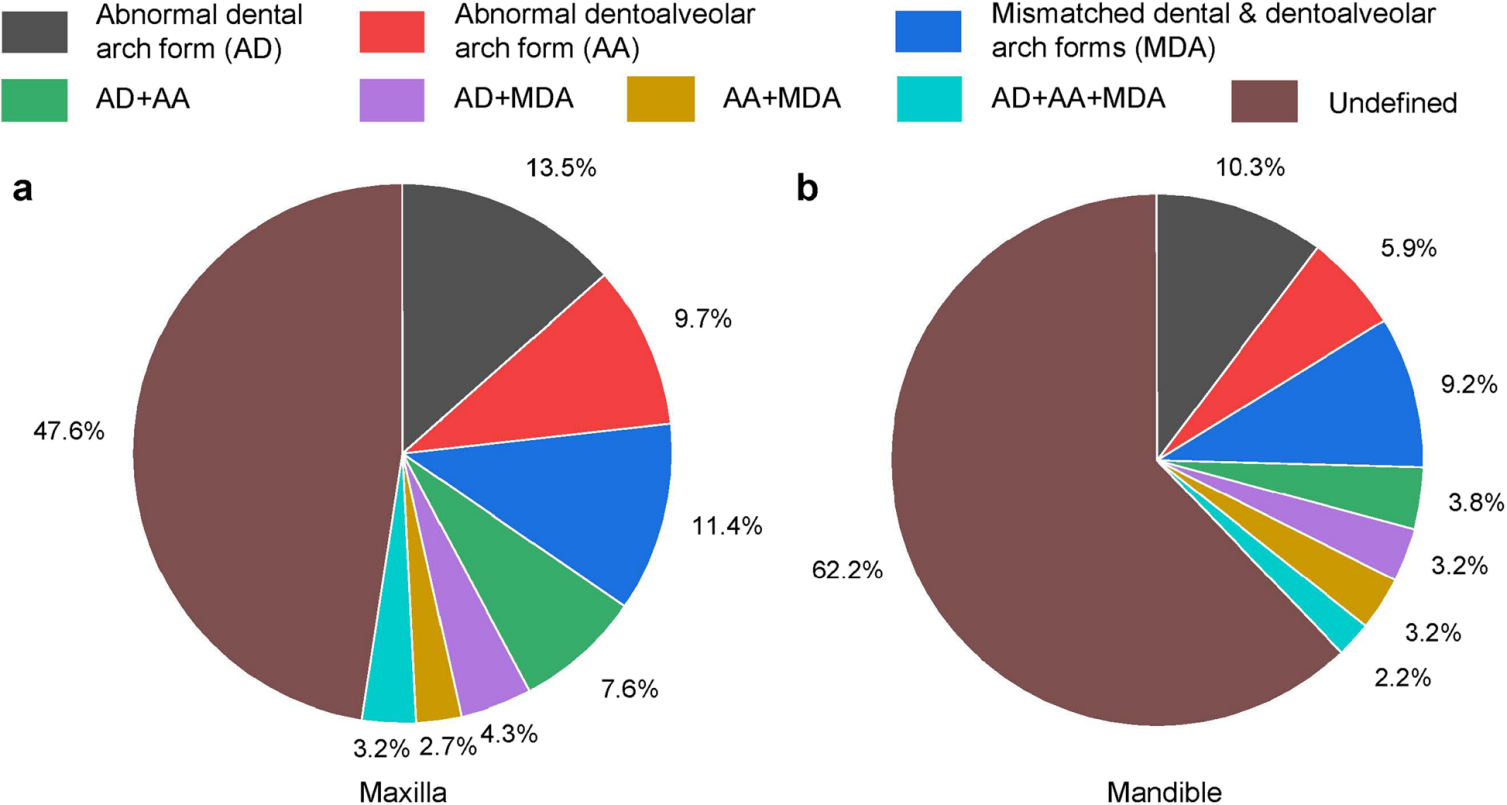
The proportion of various dental and dentoalveolar arch morphological anomalies in the maxilla (**a**) and mandible (**b**)

Based on 3 basic anomalies (AD, AA, and MDA), 4 combined anomalies were defined, namely, AD + AA, AD + MDA, AA + MDA, and AD + AA + MDA, respectively. Therefore, a total of 7 anomalies were defined, and the rest were *undefined*. The anomalies were investigated in 199 subjects with malocclusion. To estimate the likelihood or probability that a given dental/dentoalveolar arch form is anomalous, multiple rounds of anomaly detection were conducted, and the number of times detected was counted. If the count exceeds a predefined threshold, an anomaly is identified. The overall proportions of anomalies are 52.4% (Fig. 5a) and 37.8% (Fig. 5b) in the maxilla and mandible, respectively. The proportion of anomalies in the maxilla is 14.6% higher than those in the mandible. AD is the most common anomaly, accounting for 13.5% (Fig. 5a) and 10.3% (Fig. 5b) in the maxilla and mandible, respectively. MDA is the second most common anomaly after AD, accounting for 11.4% (Fig. 5a) and 9.2% (Fig. 5b) in the maxilla and mandible, respectively.

## Discussion

Orthodontists generally accept that a stable orthodontic treatment should have a coordinated individualized dental and dentoalveolar arches. Most studies suggested the use of landmarks located on the alveolar [5, 33] or basal [4] bone to predict the individualized dental arch form in permanent dentition. Since both growth (e.g., permanent tooth eruption) and external force (i.e., orthodontic force) may lead to changes in dental arch form or alveolar bone, this study investigated the association between dental and dentoalveolar arch forms, which may improve the clinical understanding of malocclusion mechanisms and assist clinicians in producing early orthodontic results consistent with the natural laws of biological variation.

Different landmarks and measurement methods may yield different results on the same dental cast [34–36]. Our results demonstrate a discernible difference between normalized dental and dentoalveolar arch forms (shapes), which is consistent with clinical observations and may be related to the buccolingual inclination and alignment of the teeth.

 Early orthodontic treatment (e.g., rapid, semi-rapid, and slow maxillary expansion) may lead to adaptive changes in the width of both upper and lower dental arches, thereby resolving mild-to-moderate crowding [37]. This study divided the normalized dental/dentoalveolar arch forms into 6 clusters, and quantified the one-to-many association between dental and dentoalveolar arch clusters in normal occlusion. These findings suggested that the dentoalveolar arch form has a certain flexibility to adapt to changes in dental arch form caused by early orthodontic treatment. Reshaping the dentoalveolar arch form may further enhance this adaptation. However, there is no consensus on the timing and regimen of early orthodontic treatment [38–42], which may be a research direction in the future.

Previous studies have shown relatively large individual variations in both dental arch dimensions [3, 12] and shapes [11, 12, 43] in normal occlusion. The dimensions of the dental arch form change continuously throughout growth and development, and the changes gradually decrease in adulthood [6]. Therefore, the expansion of the dental arch form in adults is limited [1, 3]. According to the “apical base” theory [44], the *individualized* dental arch form could be determined by WALA ridge/basal arch form [2, 3]. Our analysis of 199 pediatric patients with malocclusion illustrated that the mismatch between dental and dentoalveolar arch forms is a common morphological anomaly. This finding suggested that the goal of early orthodontic treatment may not only be the *individualized* dental arch form, but more importantly, the *individualized* matching between dental and dentoalveolar arch forms. The prediction and coordination of the *individualized* matching is a complex and comprehensive decision-making process that requires further research.

A possible limitation of this study is the sample size, though it is comparable to many previous studies. Changes in data may cause fluctuations in clustering results, consequently changing associations. In addition, our data were derived from a community survey, which may limit its representativeness of the broader population. Therefore, random sampling is recommended to alleviate this concern.

An investigation into the association between dental and dentoalveolar arch forms in different age, gender, and ethnic groups is also recommended.

## Conclusion

 Our findings based on Chengdu samples are summarized as follows:

1. There is a clear difference between dental and dentoalveolar arch forms with normal occlusion in the mixed and early permanent dentition.
2. There is a one-to-many association between dental and dentoalveolar arch forms with normal occlusion in the mixed and early permanent dentition.
3. The mismatch between dental and dentoalveolar arch forms is a common morphological anomaly in malocclusion.

## Data Availability

The data of this study are available from the corresponding authors upon reasonable request.

## Abbreviations

WALA ridge: A soft tissue band that is located above the mucogingival junction of the mandible, and below the gingival margins of mandibular tooth crowns.
LiLo curve: The smoothest projected curve depicting the layered contour of the buccal alveolar bone.
ANB: A specific measurement made on lateral cephalograms. It stands for A-point, Nasion, B-point. These points are landmarks on the X-ray image that correspond to specific anatomical structures
CBCT: Cone-Beam Computed Tomography
KNN: K-Nearest Neighbors
SVM: Support Vector Machines
NB: Naive Bayes
ND: Normalized dental arch form
NA: Normalized dentoalveolar arch form
AD: Abnormal dental arch form
AA: Abnormal dentoalveolar arch form
MDA: Mismatched dental and dentoalveolar arch forms

## References

[1] Kim K-Y, Bayome M, Kim K, Han SH, Kim Y, Baek S-H, Kook Y-A (2011). Three-dimensional evaluation of the relationship between dental and basal arch forms in normal occlusion. Korean J Orthod.

[2] Trivino T, Siqueira DF, Scanavini MA (2008). A new concept of mandibular dental arch forms with normal occlusion. Am J Orthod Dentofacial Orthop.

[3] Ronay V, Miner RM, Will LA, Arai K (2008). Mandibular arch form: the relationship between dental and basal anatomy. Am J Orthod Dentofacial Orthop.

[4] Suk KE, Park JH, Bayome M, Nam YO, Sameshima GT, Kook YA (2013). Comparison between dental and basal arch forms in normal occlusion and Class III malocclusions utilizing cone-beam computed tomography. Korean journal of orthodontics.

[5] Ball RL, Miner RM, Will LA, Arai K (2010). Comparison of dental and apical base arch forms in Class II Division 1 and Class I malocclusions. Am J Orthod Dentofacial Orthop.

[6] Carter GA, McNamara JA (1998). Longitudinal dental arch changes in adults. Am J Orthod Dentofacial Orthop.

[7] Felton JM, Sinclair PM, Jones DL, Alexander RG (1987). A computerized analysis of the shape and stability of mandibular arch form. Am J Orthod Dentofacial Orthop.

[8] de la Cruz A, Sampson P, Little RM, Artun J, Shapiro PA (1995). Long-term changes in arch form after orthodontic treatment and retention. Am J Orthod Dentofacial Orthop.

[9] Sanin C, Savara BS, Thomas DR, Clarkson QD (1970). Arc length of the dental arch estimated by multiple regression. J Dent Res.

[10] Chuck GC (1934). Ideal Arch Form*. Angle Orthod.

[11] Daou R, Nassar R, Khoury E, Ghoubril J (2020). Changes of arch form at the end of orthodontic treatment, based on the Ricketts pentamorphic arch forms. Am J Orthod Dentofacial Orthop.

[12] Omar H, Alhajrasi M, Felemban N, Hassan A (2018). Dental arch dimensions, form and tooth size ratio among a Saudi sample. Saudi Med J.

[13] Infante PF (1975). Malocclusion in the deciduous dentition in white, black, and Apache indian children. Angle Orthod.

[14] Barrow GV, White JR (1952). Developmental changes of the maxillary and mandibular dental arches*. Angle Orthod.

[15] Bishara SE, Jakobsen JR, Treder J, Nowak A (1997). Arch width changes from 6 weeks to 45 years of age. Am J Orthod Dentofacial Orthop.

[16] Defraia E, Baroni G, Marinelli A (2006). Dental arch dimensions in the mixed dentition: a study of Italian children born in the 1950s and the 1990s. Angle Orthod.

[17] Louly F, Nouer PR, Janson G, Pinzan A (2011). Dental arch dimensions in the mixed dentition: a study of Brazilian children from 9 to 12 years of age. Journal of applied oral science : revista FOB.

[18] Sillman JH (1964). Dimensional changes of the dental arches: Longitudinal study from birth to 25 years. Am J Orthod.

[19] Alexander TL, Hitchcock HP (1978). Cephalometric standards for American Negro children. Am J Orthod.

[20] Bishara SE (1981). Longitudinal cephalometric standards from 5 years of age to adulthood. Am J Orthod.

[21] Engel G, Spolter BM (1981). Cephalometric and visual norms for a Japanese population. Am J Orthod.

[22] Thilander B, Persson M, Adolfsson U (2005). Roentgen-cephalometric standards for a Swedish population. A longitudinal study between the ages of 5 and 31 years. Eur J Orthod.

[23] da Silva Filho OG, de Freitas SF, Cavassan AO (1990). Prevalence of normal occlusion and malocclusion in Bauru (Sao Paulo) students 2. Influence of socioeconomic level. Revista de odontologia da Universidade de Sao Paulo.

[24] Kondo T, Ong SH, Foong KW (2004). Tooth segmentation of dental study models using range images. IEEE Trans Med Imaging.

[25] Yuan T, Wang Y, Hou Z, Wang J (2020). Tooth segmentation and gingival tissue deformation framework for 3D orthodontic treatment planning and evaluating. Med Biol Eng Compu.

[26] Zou BJ, Liu SJ, Liao SH, Ding X, Liang Y (2015). Interactive tooth partition of dental mesh base on tooth-target harmonic field. Comput Biol Med.

[27] Wu K, Chen L, Li J, Zhou Y (2014). Tooth segmentation on dental meshes using morphologic skeleton. Comput Graph.

[28] Pepe SH (1975). Polynomial and catenary curve fits to human dental arches. J Dent Res.

[29] Ma J, Schneider L, Lapuschkin S, Achtibat R, Duchrau M, Krois J, Schwendicke F, Samek W (2022). Towards Trustworthy AI in Dentistry. J Dent Res.

[30] Luo J, Zhou L, Feng Y, Li B, Guo S (2021). The selection of indicators from initial blood routine test results to improve the accuracy of early prediction of COVID-19 severity. PLoS ONE.

[31] Liu F, Deng Y (2021). Determine the Number of Unknown Targets in Open World Based on Elbow Method. IEEE Trans Fuzzy Syst.

[32] Aminikhanghahi S, Cook DJ (2017). A Survey of Methods for Time Series Change Point Detection. Knowl Inf Syst.

[33] Gupta D, Miner RM, Arai  K, Will LA (2010). Comparison of the mandibular dental and basal arch forms in adults and children with Class I and Class II malocclusions. Am J Orthod Dentofacial Orthop.

[34] Kuntz TR, Staley RN, Bigelow HF, Kremenak CR, Kohout FJ, Jakobsen JR (2008). Arch widths in adults with Class I crowded and Class III malocclusions compared with normal occlusions. Angle Orthod.

[35] Slaj M, Spalj S, Pavlin D, Illes D, Slaj M (2010). Dental archforms in dentoalveolar Class I II and III. Angle Orthod.

[36] Uysal T, Usumez S, Memili B, Sari Z (2005). Dental and alveolar arch widths in normal occlusion and Class III malocclusion. Angle Orthod.

[37] Grassia V, d'Apuzzo F, DiStasio D, Jamilian A, Lucchese A, Perillo L (2014). Upper and lower arch changes after Mixed Palatal Expansion protocol. Eur J Paediatr Dent.

[38] Buchner HJ (1949). An Answer To Some Criticisms of Treatment Following Bicuspid Extractions*. Angle Orthod.

[39] Woon SC, Thiruvenkatachari B (2017). Early orthodontic treatment for Class III malocclusion: A systematic review and meta-analysis. Am J Orthod Dentofacial Orthop.

[40] Kluemper GT, Beeman CS, Hicks EP (2000). Early orthodontic treatment: what are the imperatives?. J Am Dent Assoc.

[41] Lopes Filho H, Maia LH, Lau TC, de Souza MM, Maia LC (2015). Early vs late orthodontic treatment of tooth crowding by first premolar extraction: A systematic review. Angle Orthod.

[42] Feres MF, Abreu LG, Insabralde NM, Almeida MR, Flores-Mir C (2016). Effectiveness of the open bite treatment in growing children and adolescents A systematic review. Eur J Orthod.

[43] Siche (1975). Oral Anatomy, 6th edition edn: Mosby.

[44] Lundström AF (1925). Malocclusion of the teeth regarded as a problem in connection with the apical base. International Journal of Orthodontia, Oral Surgery and Radiography.

